# The associations between plasma phytoestrogens concentration and metabolic syndrome risks in Chinese population

**DOI:** 10.1371/journal.pone.0194639

**Published:** 2018-03-20

**Authors:** Jie Liu, Shengquan Mi, Li Du, Xiang Li, Peiqin Li, Keyu Jia, Jing Zhao, Hong Zhang, Wenhua Zhao, Ying Gao

**Affiliations:** 1 Key Laboratory of Nutrition and Metabolism, Institute for Nutritional Sciences, Shanghai Institutes for Biological Sciences, Chinese Academy of Sciences, University of Chinese Academy of Sciences, Shanghai, China; 2 College of Biochemical Engineering, Beijing Union University, Beijing, China; 3 Institute of Biostatistics, School of Life Science, Fudan University, Shanghai, China; 4 National Institute for Nutrition and Health, Chinese Center of Disease Control and Prevention, Beijing, China; University of Hull, UNITED KINGDOM

## Abstract

Metabolic syndrome (MetS) has become an important issue in the healthcare systems of both developed and developing countries. Phytoestrogens have shown estrogenic effects, which may involve in the etiology of MetS. The current study consisted of 293 MetS cases and 264 healthy controls. The concentrations of seven plasma phytoestrogens (daidzein, genistein, glycitein, equol, enterolactone, enterodiol and coumestrol) were detected by UPLC-MS/MS. Adjusted unconditional logistic regression was used to assess the associations between plasma phytoestrogens concentration and risks of MetS, as well as the associations between plasma phytoestrogens concentration and MetS components. Linear regression was used to evaluate the associations between equol concentration in equol-producers and MetS components. Higher concentrations of total isoflavone and equol were associated with decreased risk of MetS. The equol concentration was negatively associated with waist circumference and positively associated with HDL-C level. Increased daidzein was associated with both lower waist circumference and lower fasting blood glucose levels. Our results suggested that higher plasma total isoflavone, equol and daidzein might decrease MetS risk.

## Introduction

In the past few years, obesity and some of its related disorders, including type 2 diabetes, increased triglycerides and low density lipoprotein (LDL) cholesterol/decreased high density lipoprotein (HDL) cholesterol, hypertension and cardiovascular diseases (CVD), which were referred as metabolic syndrome (MetS) [1], have become an important issue in the healthcare systems of developed and developing countries. The global prevalence of the diabetes was estimated to increase from 382 million people in 2013 to 592 million in 2035 [2].

The lower incidence of MetS in Asian populations has attracted attentions toward soy foods, which is considered as a characteristic class of food in Asian diets. There are observational studies, using food frequency questionnaires to estimate soy intakes, showed that soy intake might have protective effects on MetS or cardiovascular diseases [3, 4]. As the bioactive substances in the soy foods, phytoestrogens and its subclass isoflavone were thought to be the active ingredients of soy foods effects. Phytoestrogen is a class of non-steroidal compounds and plant products which include flavonoids, isoflavone, coumestrol and lignan. Because the structure is similar to estrogen, phytoestrogens have an affinity to estrogen receptor α and β (ERα and ERβ) [5, 6]. They bind to the estrogen receptor agonistically or antagonistically, and show some hormonal activities. Among all the phytoestrogens, dietary intakes of isoflavone and lignan have been well documented [7, 8]. The main dietary components of isoflavone are daidzein, genistein, glycitein and daidzein excretion equol. In all of these, daidzein, genistein and equol are most studied. They widely exist in vegetables, fruits, and especially soybeans [9]. The most abundant lignan, including enterolactone and enterodiol, can be found in berries, cereals, and especially flaxseed [10]. Isoflavone and lignan are metabolized by intestinal bacteria into biologically active forms after most of them are consumed as glycoside. Their binding capacity to the estrogen receptors is much lower than endogenous estrogens; but considering about their higher levels in blood, phytoestrogens can also provide a strong biological effect in our body [11]. An epidemiological study conducted in Italia showed that a 4-week phytoestrogen (genistein) supplementation decreased fasting glucose, insulin, HOMA-IR, total cholesterol, LDL-C, triglycerides body levels, and increased HDL-C and adiponectin body levels [12]. Though the exact mechanism that phytoestrogens impact on the MetS is still unclear, there are several hypotheses: First, phytoestrogens might have influence on hypothalamic neurons, which could decrease food intake and increase the effect of physical activity. Second, the ERα could increase the expression of glucose transporters on muscle cells [13]. It has been shown that phytoestrogens can lower the cancer risk of breast and prostate; phytoestrogens were also shown to have beneficial effects on cardiovascular risk factors [13–15]. Several studies have observed that phytoestrogens might improve cardiovascular parameters, such as higher HDL-C and lower body mass index (BMI) [12, 16, 17]. Meanwhile, several clinical studies reported that phytoestrogens had no significant effects on some parameters of MetS [18–20].

However, the phytoestrogens levels of these studies were estimated by food frequency questionnaires (FFQ). Due to the limitation of FFQ, there is still lack of evidence about the relationship between human body phytoestrogens levels and the risk of MetS. The favorable effects of phytoestrogens have been already taken into consideration by many countries’ Food and Drug Administration like US and China. But many other organizations thought the evidences were not enough to support the health claims. More biomarker researches are still needed.

We aimed to study the associations between blood phytoestrogens concentration and MetS risks in a Chinese population, hypothesizing that higher concentrations of phytoestrogens in blood were associated with lower risks of MetS.

## Materials and methods

### Ethics statement

The participants were informed for written consent, and the study protocol was proved by Chinese Center for Disease Control and prevention Ethical Review Committee.

### Study population and data collection

The study subjects were selected from a Chinese cohort study project called Study on Major Chronic Disease Risk Assessment System and Related Technology Developing and Application (Chinese Clinical Trial Registry number: ChiCTR-EOC-17012759). This cohort study was conducted and followed up from April 2010 to December 2012. Briefly, two rural villages and two urban neighborhood committees were selected from Beijing municipality and Zhejiang province as the study sites to recruit study subjects. All the subjects were recruited in accordance with the principle of voluntary. Individuals with serious diseases of heart, lung, liver and kidney or have limitation of motion were excluded from the study. In total, more than 7000 individuals aged from 35 to 60 years were recruited at baseline.

Information of the general characteristics of the subjects’ lifestyle, physical activity and socioeconomic status was obtained by using a structured questionnaire through interviews. Information on diet was collected by using this questionnaire. The FFQ included 11 food categories to assess dietary intake consumed during the past 6 months. But there was not enough information about the validity of the assessment to estimate phytoestrogens dietary intake using this FFQ. Height, weight, waist circumference and blood pressure were obtained by interviews, measuring by disciplined researchers. At the baseline of enrolling, EDTA anticoagulated blood was collected. Plasma was immediately separated by centrifugation (240g for 15min at 4°C) for measurement of blood chemistry such as fasting blood glucose (FBG), total cholesterol, total triglycerides and HDL-C by Hitachi 7020 chemistry analyser (Hitachi, Tokyo, Japan). Then plasma samples were stored at -70°Cfreezer until phytoestrogens measurement.

A total of 2008 participants who were recruited in Shunyi, the rural district of Beijing, were included in this study. MetS was defined according to ATPIII criteria [21]. Participants who met with 3 or more points of the following conditions were regarded as having MetS: 1) systolic blood pressure > 130 mm Hg or diastolic blood pressure > 85 mm Hg or use of antihypertensives; 2) plasma fasting glucose > 6.1 mmol/L or use of antidiabetics; 3) waist circumference (WC) > 102 cm for men or > 88 cm for women; 4) plasma total triglycerides > 1.69 mmol/L or use of lipid-lowing treatment; 5) plasma high density lipoprotein cholesterol (HDL-C) < 1.04 mmol/L for men or < 1.29 mmol/L for women. This study was a case-control study, cases were subjects who have four or more MetS factors, healthy controls were those who were free of any MetS symptoms. Controls were matched to cases on age (± 4 years). However, 29 controls had insufficient plasma sample to detect the phytoestrogens and were excluded from this study. Finally, A total of 293 MetS patients (have four or more MetS factors) and 264 age-matched (± 4 years) health controls who were with adequate plasma sample were included in this study.

### Measurements of plasma phytoestrogens concentrations

Plasma concentrations of phytoestrogens, including genistein, daidzein, glycitein, equol (a daidzein metabolite), enterolactone, enterodiol and coumestrol were evaluated by using ultra performance liquid chromatography and mass spectrum (UPLC-MS/MS). The UPLC-MS/MS method allowed accurate, sensitive and rapid analysis with plasma [22].

We calculated the coefficients of variation for quality control samples with 60 replicated samples randomly distributed among the test samples. In our study, the overall coefficients of variation were 7.8% for genistein, 8.3% for daidzein, 11.2% for glycitein, 10.2% for equol, 11.7% for enterolactone, 9.1% for enterodiol, and 10.9% for coumestrol respectively.

### Statistical analysis

The statistical analysis was performed using SAS 9.2 (SAS Institute, Cary, NC, USA). The Chi-square test for categorical variables and Wilcoxon’s rank sum test for continuous variables were used to identify differences of proportion or medians of characteristics such as sex, age, education, family income, smoking history, alcohol drinking history, BMI, waist circumference, systolic blood pressure, diastolic blood pressure, fasting blood glucose, total cholesterol, total triglyceride, HDL-C, and phytoestrogens concentration between MetS cases and controls.

The associations between phytoestrogens concentration quartiles and MetS risk were assessed by unconditional logistic regression models (adjusted for age, sex, smoking history and alcohol drinking history). ORs and 95%CIs for risk of MetS were calculated from the logistic models. Tests for trend were calculated in multiple logistic regression models by treating quartiles of phytoestrogens as continuous variables after assigning the median values in each quartile. Logistic regression was also used to assess the association between phytoestrogens concentration and risks of MetS components. MetS components were classified as dichotomized variable: hypertension (no or yes; yes was defined as blood pressure ≥ 130/85 mm Hg), waist circumference (no or yes; yes was defined as WC > 102 cm for men or > 88 cm for women), hyperglycemia (no or yes; yes was defined as fast blood glucose > 6.1 mmol/L), hyperlipidemia (no or yes; yes was defined as elevated triglycerides or low HDL-C).

We defined equol producers as plasma concentrations higher than the limit of detection, 1 ng/ml, as in previous studies [23, 24]. Among the equol producers, *β*-value and *P*-value were calculated with linear regression to estimate theassociations between equol level and MetS components (levels). All *P* values were two-sided, and statistically significance was defined as *P* <0.05.

## Results

There were 293 MetS cases and 264 not MetS controls included in this study. The characteristics of cases and healthy controls were presented in **Table 1**. No significant differences were observed on mean age, sex, education level, family yearly income, smoking, and alcohol drinking history between cases and controls.

**Table 1:** Characteristics and metabolic syndrome components levels of metabolic syndrome cases and healthy controls.

	Total (N = 557)	MetS (N = 293)	NotMetS (N = 264)	*P* ^b^
	Median (IQR)	Median (IQR)	Median (IQR)	
Age (years)	49 (44–55)	51 (47–56)	47 (42–52)	0.13
Sex, women (%)	387 (69.5%)	201 (68.6%)	186 (70.5%)	0.32
Education, n (%)				
Elementary and below	117 (21.0%)	59 (20.1%)	58 (22.0%)	0.70
Middle school	351 (63.0%)	184 (62.8%)	167 (63.3%)	
High school	75 (13.5%)	43 (14.7%)	32 (12.1%)	
College and above	14 (2.5%)	7 (2.4%)	7 (2.7%)	
Family income in previous year/yuan, n (%)^a^				
<5000	164 (29.4%)	81 (27.6%)	83 (31.4%)	0.23
5000–10000	182 (32.7%)	87 (29.7%)	95 (36.0%)	
10000–20000	154 (27.6%)	71 (24.2%)	83 (31.4%)	
>20000	29 (5.2%)	13 (4.4%)	16 (6.1%)	
Smoke history (yes/no)	116 / 441	60 / 233	56 / 208	0.86
Alcohol drinking history (yes/no)	133 / 424	68 / 225	66 / 199	0.74
Height (cm)	159 (154–165)	159 (155–167)	158 (154–163)	0.07
Weight (kg)	65.7 (56.5–75.5)	74 (68–82)	56.5 (52.2–62.0)	<0.01
BMI (kg/m^2^)	25.8 (22.9–29.0)	28.7 (27.2–30.6)	22.8 (21.3–24.1)	<0.01
Waist circumference (cm)	84.6 (74.6–94.4)	93.7 (88.9–100.5)	74.3 (70.6–78.4)	<0.01
Systolic blood pressure (mm Hg)	124 (114–140)	140 (130–150)	116 (110–120)	<0.01
Diastolic blood pressure (mm Hg)	82 (78–92)	92 (86–100)	78 (74–82)	<0.01
Fasting blood glucose (mmol/l)	5.2 (4.8–5.9)	5.9 (5.2–6.7)	5.0 (4.6–5.2)	<0.01
Total cholesterol (mmol/l)	5.2 (4.6–5.8)	5.4 (4.8–6.1)	4.9 (4.4–5.4)	<0.01
Total triglyceride (mmol/l)	1.4 (0.8–2.4)	2.4 (1.8–3.4)	0.8 (0.6–1.0)	<0.01
HDL-C (mmol/l)	1.3 (1.1–1.6)	1.1 (1.0–1.2)	1.6 (1.4–1.9)	<0.01
Equol producer (n)	437	219	218	

IQR: interquartile range; BMI: body mass index; HDL-C: high-density lipoprotein cholesterol.

MetS met with 4 or more points of the following: systolic blood pressure >130 mm Hg or diastolic blood pressure > 85 mm Hg or use of antihypertensives; plasma fasting glucose > 6.1 mmol/L or use of antidiabetics; waist circumference > 102 cm for men or > 88 cm for women; plasma total triglycerides > 1.69 mmol/L or use of lipid-lowing treatment; plasma HDL-C < 1.04 mmol/L for men or < 1.29 mmol/L for women

^a^ Missing values existed.

^b^ *P* values were calculated by Chi-square test or Wilcoxon’s rank sum test.

The plasma phytoestrogens median levels in cases and controls were presented in **Table 2**. Total isoflavone was calculated as the sum of daidzein, genistein, glycitein and equol; and total lignan was calculated as the sum of enterolactone and enterodiol. The median plasma concentrations of equol (1.9 ng/ml for cases and 3.2 ng/ml for controls; *P* = 0.002), enterolactone (0.5 ng/ml for cases and 0.6 ng/ml for controls; *P* = 0.001), enterodiol (3.4 ng/ml for cases and 4.9 ng/ml for controls; *P* = 0.019), coumestrol (2.6 ng/ml for cases and 2.7 ng/ml for controls; *P* = 0.003) and total lignan (4.0 ng/ml for cases and 6.5 ng/ml for controls; *P* = 0.001) were significantly different between cases and controls (*P*<0.05). The median concentrations of daidzein, genistein, glycitein, and total isoflavone were higher in controls than in cases, but there was no statistically significant difference between these two groups.

**Table 2:** Plasma concentration of phytoestrogens between cases and controls.

Phytoestrogens ng/ml	MetS (N = 293)	Not MetS (N = 264)	*P* ^c^
	Median (IQR)	Median (IQR)	
Daidein	46.9 (31.7–84.6)	47.6 (29.9–82.6)	0.78
Genistein	99 (68.7–168)	112 (71.7–220)	0.14
Glycitein	5.4 (3.8–8.5)	5.7 (3.9–9.3)	0.46
Equol	1.9 (1.0–12.2)	3.2 (1.0–44.9)	0.002
Total isoflavone^a^	176 (114–331)	196 (116–393)	0.21
Enterolactone	0.5 (0.2–1.1)	0.6 (0.2–1.7)	0.001
Enterodiol	3.4 (1.2–8.9)	4.9 (2.1–13.5)	0.019
Total lignan^b^	4.0 (1.6–10.4)	6.5 (2.6–15.6)	0.001
Coumestrol	2.6 (2.2–2.9)	2.7 (2.3–3.0)	0.003

IQR: Inter quartile range

^a^ Total isoflavone was calculated as the sum of daidzein, genistein, glycitein and equol.

^b^ Total lignan was calculated as the sum of enterolactone and enterodiol.

^c^ *P* value was calculated by Wilcoxon test.

In all the participants, a decreasing trend in the risk of MetS was found when total isoflavone concentration increased (*P* for trend = 0.017), and a marginally trend for equol concentration (*P* for trend = 0.05) (**Table 3**). The individuals in the highest quartile of total isoflavone concentration had a lower risk of MetS compared with the lowest quartile (OR = 0.62; 95%CI: 0.41, 0.90); the individuals in the highest quartile of equol had a marginally lower risk of MetS (OR = 0.70, 95%CI: 0.46, 0.91); the highest quartile of total lignan was associated with increased risk of MetS (OR = 1.35, 95%CI: 1.13, 1.73) when compared to the lowest quartile. There was no significant association between the risk of MetS and the plasma concentrations of other phytoestrogens (daidzein, genistein, glycitein, enterolactone, enterodiol, and coumestrol).

**Table 3:** Associations between plasma phytoestrogens concentration and metabolic syndrome risks.

	Quartiles of phytoestrogens concentration							*P* for trend
	Q1	Q2		Q3		Q4		
	OR	OR	95%CI	OR	95%CI	OR	95%CI	
Daidein	1.00	0.95	0.55–1.49	0.84	0.53–1.24	0.72	0.48–1.07	0.13
Genistein	1.00	0.58	0.48–1.05	0.80	0.55–1.22	0.91	0.65–1.48	0.19
Glycitein	1.00	0.77	0.48–1.07	0.67	0.45–1.01	0.72	0.46–1.05	0.65
Equol	1.00	0.98	0.90–1.25	0.85	0.66–1.15	0.70	0.46–0.91	0.05
Total isoflavone^a^	1.00	0.90	0.66–1.28	0.74	0.48–1.07	0.62	0.41–0.90	0.017
Enterolactone	1.00	1.42	1.02–2.02	1.36	0.93–1.97	1.24	0.90–1.79	0.58
Enterodiol	1.00	0.89	0.57–1.24	1.07	0.83–1.31	1.16	0.98–1.48	0.64
Total Lignan^b^	1.00	1.01	0.96–1.36	1.15	0.94–1.53	1.35	1.13–1.73	0.10
Coumestrol	1.00	0.74	0.51–1.18	0.98	0.79–1.36	1.00	0.86–1.27	0.14

Values were adjusted for age, sex, education, income, smoking history, and alcohol drinking history.

All results were accessed by logistic regression.

^a^ Total isoflavone was calculated as the sum of daidzein, genistein, glycitein, and equol.

^b^ Total lignan was calculated as the sum of enterolactone and enterodiol.

Statistically significant negative association between the equol concentration and waist circumference was observed among the equol producers (*β*-value = -1.28, *P* = 0.03). Compared with the lowest quartile of equol concentration, the waist circumference decreased more than 3cm in the highest quartile. There was a positive association between plasma equol concentration and HDL-C (*β*-value = 0.05, *P* = 0.04) (**Table 4**).

**Table 4:** Associations between plasma equol concentration in equol producers and metabolic syndrome components.

	Q1 ^a^ 1–1.5 ng/ml	Q2 ^a^ 1.5–4.4ng/ml	Q3 ^a^ 4.4–39.3ng/ml	Q4 ^a^ 39.3–578.2ng/ml	*β*-value	*P* for trend
MetS/Not MetS (n/n)	50 / 55	70 / 45	57 / 51	43 / 68		
Fasting blood glucose (mmol/l)	5.5 (5.2–5.7)	6.3 (5.9–6.7)	5.5 (5.2–5.7)	5.8 (5.4–6.3)	0.01	0.35
Waist circumference (cm)	85 (82–87)	89 (86–90)	86 (83–88)	81 (79–83)	-1.28	0.03
Total cholesterol (mmol/l)	5.2 (5.0–5.4)	5.3 (5.1–5.5)	5.3 (5.1–5.4)	5.1 (4.9–5.3)	-0.03	0.12
Total triglyceride (mmol/l)	1.8 (1.5–2.1)	2.4 (2.0–2.8)	2.1 (1.6–2.5)	1.4 (1.2–1.6)	-0.15	0.06
HDL-C (mmol/l)	1.4 (1.3–1.5)	1.2 (1.2–1.3)	1.4 (1.3–1.4)	1.5 (1.4–1.5)	0.05	0.04
SBP (mm Hg)	126 (123–130)	131 (128–135)	130 (124–132)	123 (119–126)	-1.0	0.26
DBP (mm Hg)	84 (82–86)	86 (84–88)	86 (84–88)	82 (80–85)	-0.6	0.18

SBP: Systolic blood pressure; DBP: Diastolic blood pressure; HDL-C: High density lipoprotein cholesterol

All values were assessed by linear regression

^a^ All the MetS components levels were shown as mean (95%CI)

**Fig 1:**
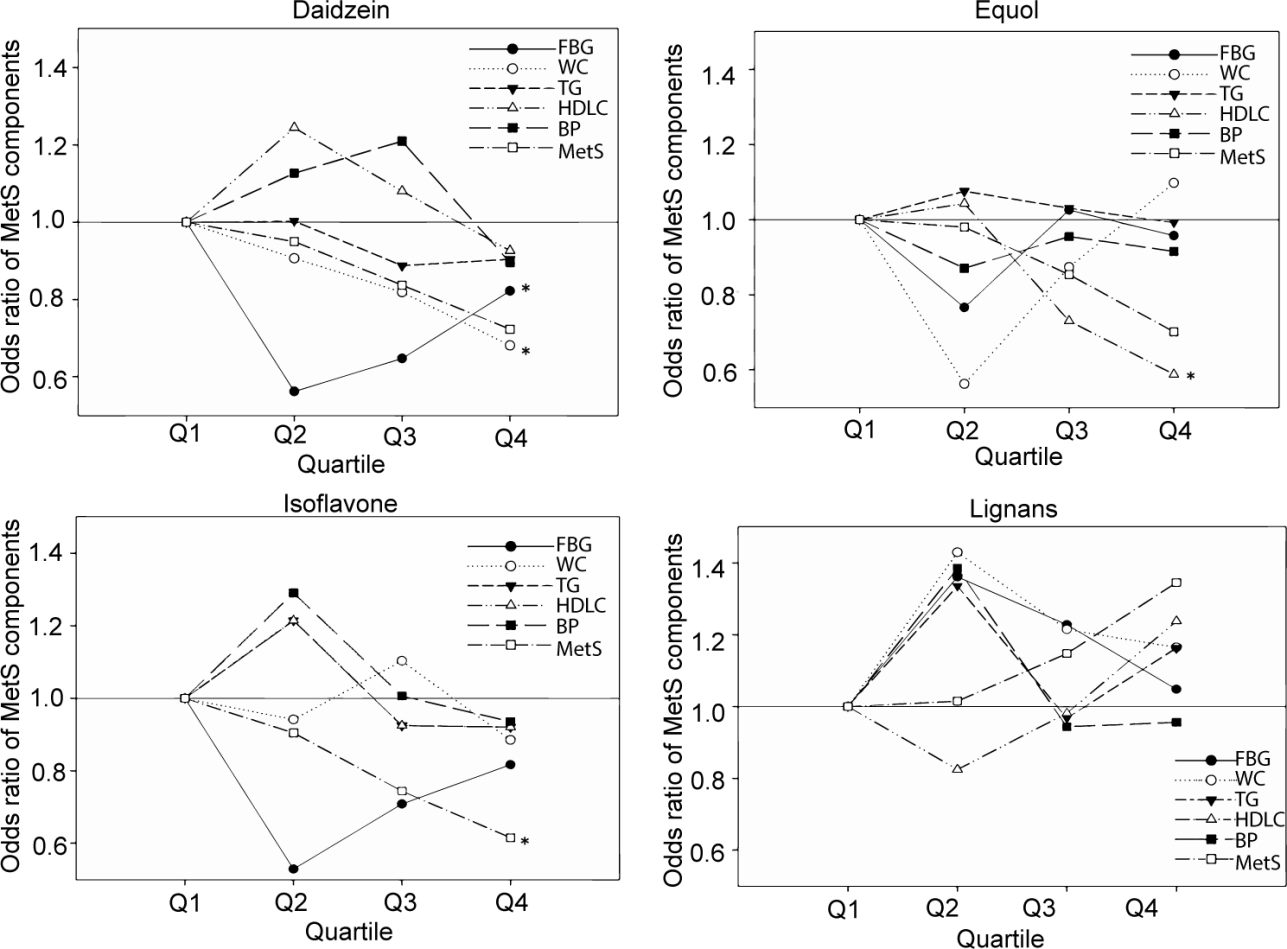
Associations between subclasses of the phytoestrogens and metabolic syndrome components risks. Odds ratios were obtained from logistic regression. Cases were defined as following: BP: systolic blood pressure > 130 mm Hg or diastolic blood pressure > 85 mm Hg or use of antihypertensives; FBG: plasma fasting glucose > 6.1 mmol/L or use of antidiabetics; WC: waist circumference > 102 cm for men or > 88 cm for women; TG: plasma total triglycerides > 1.69 mmol/L or use of lipid-lowing treatment; Low-HDL-C: plasma HDL-C < 1.04 mmol/L for men or < 1.29 mmol/L for women. * *P* for trend < 0.05.

The associations between all the phytoestrogens quartile concentration and MetS components risks were presented in **Fig 1**. A decreasing trend was found in the risk of waist circumference with increasing daidzein (*P* for trend = 0.016). When compared to the lowest quartile, daidzein had a negative association with risk of fasting blood glucose, though a rising trend according to the quartile was observed (*P* for trend = 0.013). Higher equol concentration was associated with decreased risk of low HDL-C (*P* for trend = 0.036), and the OR for the highest quartile compared to the lowest quartile was 0.59 (95%CI: 0.36, 0.82).

## Discussion

In this case-control study of plasma phytoestrogens concentration and MetS risks in Chinese population, we found a statistically significant negative association between plasma total isoflavone concentration and MetS risks. The individuals with higher total isoflavone concentration had a lower risk of MetS. In all the equol producers, we observed statistically significant association between plasma equol concentration and MetS components (waist circumference and HDL-C). We also observed a decreasing trend between plasma daidzein concentration and the risk of waist circumference, and an increasing trend between fasting blood glucose and daidzein.

So far, most studies researched the relationship between single MetS components and phytoestrogens concentration using dietary questionnaires to estimate phytoestrogens intake [3, 4, 25–31]. Only one case-control study presented the relationship between plasma isoflavone and diabetes, the results showed that plasma isoflavone concentration was associated with decreased risk of type 2 diabetes in Korean women [32].

Phytoestrogens and estrogen have similar chemical structure, thusphytoestrogens have an affinity for estrogen receptor α and β and other receptors such as peroxisome proliferator-activated receptor (PPAR) family receptors [33, 34] and the arylhydrocarbon receptor (AhR) [34, 35]. The estrogen receptor and the other receptors are known to regulate numerous biological activities, such as glucose metabolism, lipid metabolism and cardiovascular efficiency [36–38]. Some experimental studies showed that phytoestrogens had glucose regulating and anti-obesity effect [39, 40]. A randomized controlled trial using genistein supplementation intervention showed that genistein decreased the risks of diabetes and cardiovascular diseases in postmenopausal women with MetS [12]. By increasing the secretion of insulin and suppressing the proliferation of islet cell, phytoestrogens also had an effect on decreasing glucose [41].

Several epidemiological studies have evaluated the association between phytoestrogens intake and the incidence of MetS. A study conducted in Framingham showed that higher intake of phytoestrogens in postmenopausal women appeared to be associated with a favorable metabolic cardiovascular risk profile [3]. A Shanghai women’s health study reported that the highest quantile of soybean intake exhibited a significantly reduced risk (47%) of type 2 diabetes compared with the lowest quintile [31]. However, evidences on the association between soybean intake and type 2 diabetes was inconsistent. A population-based cohort study in Japan reported that the intake of soybean products and isoflavones were associated with a lower risk of type 2 diabetes in overweight women, but no association was found in men or normal-weight women [28].

Although the effect of phytoestrogens on MetS was relatively clear from experimental studies, the results from epidemiological studies were inconsistent [3, 4, 25–31]. The divergences might result from geographic differences of vegetable food consumption. It was reported that the serum isoflavone concentration in Asian population was ten times higher than that in western population [32]. Furthermore, previous studies used FFQs to calculate the intake of phytoestrogens, these methods had a highly sensitivity to measurement error. Besides, the inconsistent results might also be caused by the failure of distinguishing equol producers and non-producers. The metabolism of phytoestrogens, especially isoflavone, was different in equol producers and non-producers. Equol was biotransformed from daidzein by gut bacteria, about 80% of Asian individuals produced equol after consuming soy products [42, 43]. The methods of FFQs couldn’t differentiate equol producers and non-producers.

In our study, we found that plasma total isoflavone concentration was associated with MetS risks in Chinese population. This might be explained by the role of estrogen in the development of MetS. Except for the affinity to estrogen receptor, isoflavone could also act as androgen pathway modulators. Depending on the endogenous estrogen levels, isoflavone could play both agonist and antagonist roles to estrogen receptor [44]. It was also found that in equol producers the concentration of equol had a negative association with waist circumference and a positive association with HDL-C. These results came from all the equol producers suggested that equol had a protective effect on the components of MetS, which could be explained by the specific bioactivity of equol. Equol had 100-fold higher affinity for estrogen receptors and higher bioactivity [45]. It also had effect on slowing plasma clearance rate. Besides, we found the higher concentration of daidzein might decrease waist circumference and increase the fast blood glucose. The result of fast blood glucose increasing in our study was different from previous studies. This might be resulted from the multiple effects of isoflavone, which acted as the both agonist and antagonist of estrogen receptor [10]. In our study, higher total lignan was associated with increased risk of MetS and MetS components. These results were consisted with previous study reporting that the total lignan was associated with higher TG and blood glucose [46]. Total lignan might affect the sex hormones levels which were vital risk factors for MetS, and also involve in impaired lipid profiles metabolism and insulin resistance [46, 47].

The first advantage of this study was the direct measurement of plasma phytoestrogens concentration, which could reflect the absorption and metabolism of phytoestrogens in human body. Secondly, we selected the case and control within the same cohort, which could avoid the selection bias. Our study had several limitations. First of all, our study was a case-control study and might be subject to a reverse causality. However, some phytoestrogens supplement studies reported that long-term intake of soy protein could improve blood lipid profiles and decreased blood pressure [48, 49]. What’s more, appreciable variation in plasma phytoestrogens according to the counts of the MetS components (from 4 to 5) was not observed in this study. Further prospective study is needed to confirm our results. Secondly, we could not access the available data of dietary intake of phytoestrogens due to the lack of food questionnaires. Therefore, we could not evaluate whether the high levels of plasma phytoestrogens were mainly influenced by high dietary intake. Thirdly, phytoestrogens had short lifetime in the blood, and the plasma concentration might be affected by the last meal before blood drawing. To minimize the effect of meals, we collected whole blood after a 10–12 hour overnight fast.

In summary, the total isoflavone concentration was associated with a decreased risk of MetS in Chinese population. In equol producers, the equol concentration had negative association with waist circumference and a positive association with HDL-C. Larger intervention study is needed to verify the modifying effect of phytoestrogens on MetS.

## Supporting information

S1 File Questionnaire of the current study. (PDF)
