# Supplementary material for: The associations between plasma phytoestrogens concentration and metabolic syndrome risks in Chinese population
Source: PLoS One. 2018 Mar 20;13(3):e0194639. doi: 10.1371/journal.pone.0194639 (PMC5860756; doi:10.1371/journal.pone.0194639)
Supplement: S1 File — (PDF) [file pone.0194639.s001.pdf]

“十一五”国家科技支撑课题重要慢性病风险评估体系与干预适宜技术及应用

调查表

调查对象的基本信息
姓 名:________________________
身份证号：____________________
家庭住址:______________________
联系电话: __________调查日期________

第一部分 一般信息
A1.性别
①男 ②女□
A2．出生日期_____年_____月 ______日（公历） □□□□/□□/□□
A3．民族
①汉族 ②壮族 ③满族 ④回族 ⑤苗族
⑥维吾尔族 ⑦彝族 ⑧土家族 ⑨蒙古族 ⑩藏族 〇11 其他
A4．文化程度
①文盲、半文盲 ②小学 ③初中 ④高中或中专 ⑤大专及大专以上
A5．主要职业
①农林牧渔水利业生产人员 ②生产、运输设备操作人员及有关人员
③商业、服务业人员 ④国家机关、党群组织、企业、事业单位负责人
⑤办事人员和有关人员 ⑥专业技术人员 ⑦军人 ⑧其他劳动者
⑨在校学生 ⑩未就业 〇11 离退休人员
A6.你现在的婚姻状况是
① 未婚 ②在婚 ③离异 ④丧偶
A7．家庭人均年收入____（元）
第二部分 健康信息
糖尿病
B1.你知道哪些人容易得糖尿病吗？（多选。不念出答案，1=是，2=否；）
①年龄 45 岁以上
②经常不活动的人
③超重或肥胖的人
④有糖尿病家族史者
⑤怀孕时曾患糖尿病者
⑥曾生育过巨大儿（4000 克/8 斤以上）的女性
⑦高血压患者
⑧心脑血管病变者
⑨血脂异常
B2.有医生诊断过你患糖尿病吗？
① 是，是____年
② 否
③ 不知道
其他疾病
除了糖尿病，你有没有被乡镇/社区级或以上医院的医生诊断患有以下疾病？

疾病	患病情况（1=是，2=否）	诊断时间（年）	近两周是否服用降压药（1=是，2=否）
B3 高血压			
B4 冠心病			
B5 脑卒中			
B6 心肌梗死			
B7 慢性呼吸系统疾病			

（如慢支、肺气肿、哮喘）			
B8 恶性肿瘤			

（诊断时间不清楚，填写“9999” ）

第三部分 家族史（1=是，2=否，9=不清楚或者没有兄弟/姐妹）

疾 病	父亲	母亲	兄弟/姐妹
C1 肥胖			
C2 糖尿病			
C3 高血压			
C4 冠心病			
C5 脑卒中			
C6.恶性肿瘤			

第四部分 吸烟与饮酒

吸烟情况

D1. 到目前为止，合计起来你是否至少吸过 100 支烟或 2 两烟叶？

①是 ②否

D2. 你现在或最近吸烟吗？

① 是 ② 否

你现在平均每天吸烟______支（1 两烟叶折合 50 支香烟）

D3.目前，你工作或生活的环境里，是否有人当着你的面吸烟？

①是 ②否

饮酒情况

D4．最近 1 年内，你至少每周喝一次酒吗？（包括任何种类的酒）

① 是

② 否

第五部分 饮食情况

E1. 你认为脂肪是保持健康必需的吗？

（脂肪指烹调油、肥肉、干果等所有食物里的油脂）

①是②不是③不知道

E2. 你知道成人每人每天烹调用油的量应该不超过多少吗？

① 知道

② 不知道

应该不超过 克？（不清楚填“999”）

E3. 你认为有必要控制烹调用油的量吗？

① 全有必要②有必要③一般④不必要⑤根本没必要

E4. 下列哪一种食物不含胆固醇？

①花生油②鸡蛋③羊肉④带鱼⑤以上都不是⑥不清楚

E5．你知道成人每日摄入胆固醇应不超过多少吗？ □

① 知道

② 不知道

应该不超过 毫克？（不清楚填“999”）
E6．你希望了解常见食物胆固醇的含量吗？
① 希望②希望③一般④不希望⑤很不希望
E7. 你一般每天吃 顿饭？
E8 早餐习惯 你一般每周在家吃___次早餐
你一般每周在餐馆吃___次早餐
你一般每周在食堂吃___次早餐
E9 午餐习惯 你一般每周在家吃___次午餐
你一般每周在餐馆吃___ 次午餐
你一般每周在食堂吃 ___次午餐
E10 晚餐习惯 你一般每周在家吃 次晚餐
你一般每周在餐馆吃__ 次晚餐
你一般每周在食堂吃 __次晚餐
E11 你家通常在一起吃饭的有 __个人?
其中 6 岁及以下一起吃饭的有__个人?
下面请你回忆过去半年里各种食物的消费情况。

食物名称	是否吃			进食次数		平均每次食用量
	1=是 2=否	次/天	次/周	次/月	次/年	
E12 主食（米、面、杂粮）						
E13 薯类（红薯\山药\芋头\土豆等）						
E14 油炸面食（油条＼油饼等）						
E15 猪、牛、羊、禽肉、内脏						
E16 水产品						
E17 奶及奶制品						
E18 蛋类						
E19 豆腐及豆制品						
E20 干豆类						
E21 新鲜蔬菜						
E22 新鲜水果						
以下以家庭为单位（不清楚用量的填99.9）	全家每月消费量					
E23 植物油（斤）						
E24 动物油（斤）						
E25 盐（克）						

第六部分 身体活动情况
F1 你知道成人每天应该活动多少步吗？
① 知道

② 不知道

应该活动__步？

F2 在过去 12 个月里，你干过农活吗？

① 干过，有农忙、农闲之分

② 干过，但没有农忙、农闲之分

③ 没有

一年里，农忙的季节有__个月？

F3 通常的业余时间里，你至少每周锻炼一次吗？（不包括步行或骑自行车）

① 是

② 否

F3_1 你每周锻炼 __次？

调查结束时间：□□时：□□分

调查员签名

审核员签名

National 11th Five-Year Technology Support Programs--Study and Application on Risk Assessment System and Intervention Appropriate Technique of Main Chronic Diseases

Questionnaire

Basic information of the participants

Name：____

ID number： ___________________________________

Home Address：______________________________________

Phone： __________________Date of Survey_____________

Part I Basic information

A1.Sex

① male ②female

A2．Birthdate _____year_____month ______day □□□□/□□/□□

A3．Nationality

① Han ②Zhuang ③Man ④Hui ⑤Miao

⑥Weiwuer ⑦Yi ⑧Tujia ⑨Menggu ⑩Zang ⑪Other

A4．Education

①Illiterate, semi-literate ②primary school ③junior high school ④High School or Technical School ⑤College graduate

A5．Principal Occupation

① Agriculture, forestry, fishing and fishery production personnel ②Production, transportation equipment operators and related personnel③Commercial and service personnel④Heads of state organs, party organizations, enterprises and institutions⑤ official staff and personnel ⑥Technical personnel⑦soldier ⑧other ⑨student ⑩ unemployed ⑪ retiree

A6. Your current marital status is

② unmarried ②married ③divorced ④Death of a spouse

A7．Family income per capita each year____ （RMB）

Part 2 Health

Diabetes mellitus

B1. Do you know who is prone to diabetes?(Multiple Choices. 1= yes, 2= no)

① over 45 years old
② inactive person
③ overweight or obese person
④ family history of diabetes
⑤ mother had diabetes when pregnant
⑥ have had a huge baby (400g / 8kg)
⑦ patients with hypertension
⑧ patients with cerebral vascular diseases
⑨ dyslipidemia

B2. Has the doctor diagnosed you with diabetes?

① yes
② no
③ don't know

Other diseases

In addition to diabetes, have you ever been diagnosed with the following diseases in a township/community level or above hospital?

Disease	Status (1 = yes, 2= no)	Diagnosis of time (year)	Have you taken blood pressure medication in the past two weeks (1 = yes, 2= no)

B3 Hypertension			
B4 Coronary heart disease (CHD)			
B5 Stroke			
B6 Myocardial infarction			
B7Chronic respiratory disease			
B8 Malignant tumor			

(if the time at diagnosis is not clear, fill in "9999")

Part 3 Family history (1 = yes, 2= no, 9= unclear or no brothers/sisters)

Disease disease	father	mother	Brother/sister
C1 Obesity			
C2 Diabetes			
C3 Hypertension			
C4 Coronary heart disease (CHD)			
C5 Stroke			
C6. Malignant tumor			

Part 4 smoking and drinking

Smoking status

D1. Up to now, do you smoke at least 100 cigarettes or 100 g tobacco?

①yes ②no

D2. Do you smoke now or recently?

①yes ②no

D3. Currently, do you work or live in an environment where someone smokes in front of you?

①yes ②no

Drinking status

D4． In recent 1 year, do you drink wine at least once a week?(including any kind of wine)

① yes ②no

Part 5 Dietary

E1. Do you think that fat is essential for health?

(fat in cooking oil, meat, dried fruit and other foods all grease)

① yes②no③don't know

E2. Do you know how much oil should an adult intake everyday

①Yes, I know

②Don't know

Should not exceed___grams? (fill "999" if you are not clear)

E3. Do you think it is necessary to control the amount of cooking oil?
①It is absolutely necessary②it is necessary③general④unnecessary⑤absolutely unnecessary

E4. Which of the following food do not contain cholesterol?
①Peanut oil②egg③mutton④hairtail⑤None of the above⑥don't know

E5. Do you know how much cholesterol should be intake a day?
①I know
②Don't know

No more than ___ mg (fill "999" if you are unclear)

E6. Do you want to know how much cholesterol in common food?
① absolutely yes②yes③general④no⑤absolutely no

E7. How many meals do you have every day?____

E8 Breakfast
How many times do you usually eat a breakfast at home every week?______
How many times do you usually eat a breakfast in restaurant every week?______
How many times do you usually eat a breakfast in dining room every week?______

E9 lunch
How many times do you usually eat a lunch at home every week?______
How many times do you usually eat a lunch in restaurant every week?______
How many times do you usually eat a lunch in dining room every week?______

E10 Dinner
How many times do you usually eat a Dinner at home every week?______
How many times do you usually eat a Dinner in restaurant every week?______
How many times do you usually eat a Dinner in dining room every week?______

E11 How many people do you have dinner with?_______
How many of them are under 6 years old?__________

Will you please recall of food consumption in the past half a year.

Food	Whether or not to eat	Eating times				The average Consumption each time
	1=yes2=no	Time/day	Time/week	Time/month	Time/year	
E12 staple food (rice,noodles,grains)						
E13 potato (sweet potatoes,yams, taro, potato, etc.)						
E14 Fried noodles, Fried dough sticks, oil cake, etc.)						
E15 pigs, cattle, sheep, poultry, liver						
E16aquatic products						
E17 milk and dairy products						

E18 eggs						
E19 tofu and soy products						
E20 dry beans						
E21fresh vegetables						
E22 fresh fruit						
The following as a family unit(fill “999” if it is not clear)	The whole family consumption per month					
E23 vegetable oil(500g)						
E24 animal fat(500g)						
E25 salt(g)						

Part 6 Physical activity

F1 Do you know how much activity should an adult do every day?

① Yes, I know. It is equal to walk ________ steps.

② I don’t know

F2 Have you done farm work in the last 12 months?

① Have done, have agricultural busy, agricultural idle point

② Have done, don’t have agricultural busy, agricultural idle point

③ No

In a year, how many months were the busy farming time?____

F3 In the spare time, do you exercise every week?

① yes

② no

F3_1 How many times do you exercise every week?______

Investigation end time:____________

Investigator signature:_______

The examinant signature:________
